# Synthesis and Conformation of Substituted Chiral Binaphthyl-Azobenzene Cyclic Dyads with Chiroptical Switching Capabilities

**DOI:** 10.3390/molecules16021603

**Published:** 2011-02-14

**Authors:** Kazuto Takaishi, Masuki Kawamoto

**Affiliations:** Supramolecular Science Laboratory, RIKEN, 2-1 Hirosawa, Wako, Saitama 351-0198, Japan

**Keywords:** optical rotation, circular dichroism, axial chirality, helical chirality, binaphthyl, azobenzene, DFT calculation, chiroptical switch

## Abstract

Optically active binaphthyl-azobenezene cyclic dyads were synthesized to develop a photochromic switching molecule. Azobenezene moieties were *cis*-*trans* isomerized by photoirradiation. As a reflection of the structural change, the specific optical rotation and circular dichroism underwent significant shifts. Under certain conditions, the positive-negative and zero-positive (or zero-negative) signals were reversed. Optical rotation may potentially be applied in noise-cancelling nondestructive photoswiches. The conformations were studied by experimental and theoretical methods. The results revealed that the helical chirality, (*P*) or (*M*), of the *cis*-azobenzene moiety was induced by intramolecular axial chirality. The twist direction depended on the axial chirality as well as the azobenzene linkage position to the binaphthyls, but was independent of the identity of substituted groups. 2,2’-Linked-(*R*)-binaphthyl was found to induce *cis*-(*P*)-azobenzene, whereas symmetrically 7,7’-linked-(*R*)-binaphthyl was found to induce *cis*-(*M*)-azobenzene.

## 1. Introduction

Molecular scaffolds designed for disparate purposes may be linked to enhance the properties of one component or to confer altogether new properties. Stimulus-driven chiroptical switches such as light-, electrically-, pH-, ion-, and temperature-driven switches have received a great deal of attention in the past decade [1,2,3,4,5,6,7,8,9,10,11]. Their high sensitivity and fast response are of great interest for molecular devices. These molecular switches are expected to find application in the fields of noise cancellation data storage, display instrument, and modulator.

Meanwhile, the axially chiral 1,1’-binaphthyl skeletons represented by BINOL [12,13] and BINAP [14,15] have a wide, flexible asymmetric field. Therefore, binaphthyl skeletons have made great contributions to the fields of catalytic asymmetric synthesis [14,15,16,17,18,19,20,21,22,23,24,25,26,27,28], specific molecular recognition [29,30,31,32,33,34,35,36,37,38,39,40,41], helical twisting of liquid crystals [42,43,44,45,46,47,48,49,50,51,52,53,54,55], and computational chemistry [56,57,58,59,60,61,62]. Azobenzenes are frequently used as photochromic components due to their switchable absorption spectra, fluorescence properties, and association constants [63,64,65,66,67,68,69,70,71,72,73,74,75,76,77,78,79,80,81,82,83,84,85,86]. The *cis* and *trans* forms of the azobenzene skeleton differ significantly in length. Therefore, it was thought that the combination of binaphthyls and azobenzenes may lead to novel molecular switches with optical properties that are derived from the chirality of the components.

**Figure 1:**
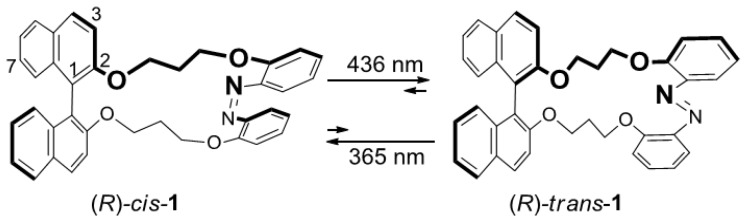
Photoisomerization of binaphthyl-azobenzene dyads **1**.

Previously, Kawamoto *et al*. described a basic binaphthyl-azobenzene dyad **1** (Figure 1) and its enantiomer [87]. The azobenzene moiety of **1** was efficiently photoisomerized to yield switching behavior, as observed in the intensity of the circular dichroism (CD) and the helical twisting power (HTP) against liquid crystal materials.

We have continued this work to analyze the photoswitchable optical properties, including absorption, CD, and optical rotation, using substituted binaphthyl-azobenene dyads and their regioisomers [88,89]. Above all, we focused on the switching of the optical rotation, which can be detected at an unabsorbed wavelength, so that the target compounds did not degrade during measurements.

**Figure 2:**
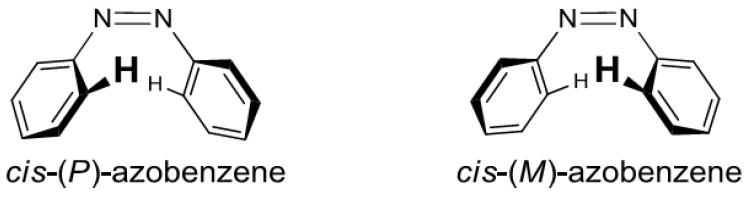
Helicity of the *cis*-azobenzenes.

The *cis*-azobenzene isomers assume an inherent helicity, (*P*) or (*M*), caused by the steric hindrance of each of the two benzene rings (Figure 2). The properties of the helicity were not well understood with a notable exception [90], so we proceeded to investigate the twisting patterns of the *cis*-azobenzene moieties induced by intramolecular chirality transfer from the axial chirality of the binaphthyl units.

## 2. Results and Discussion

### 2.1. Binaphthyl-Azobenzene Cyclic Dyads

**Figure 3:**
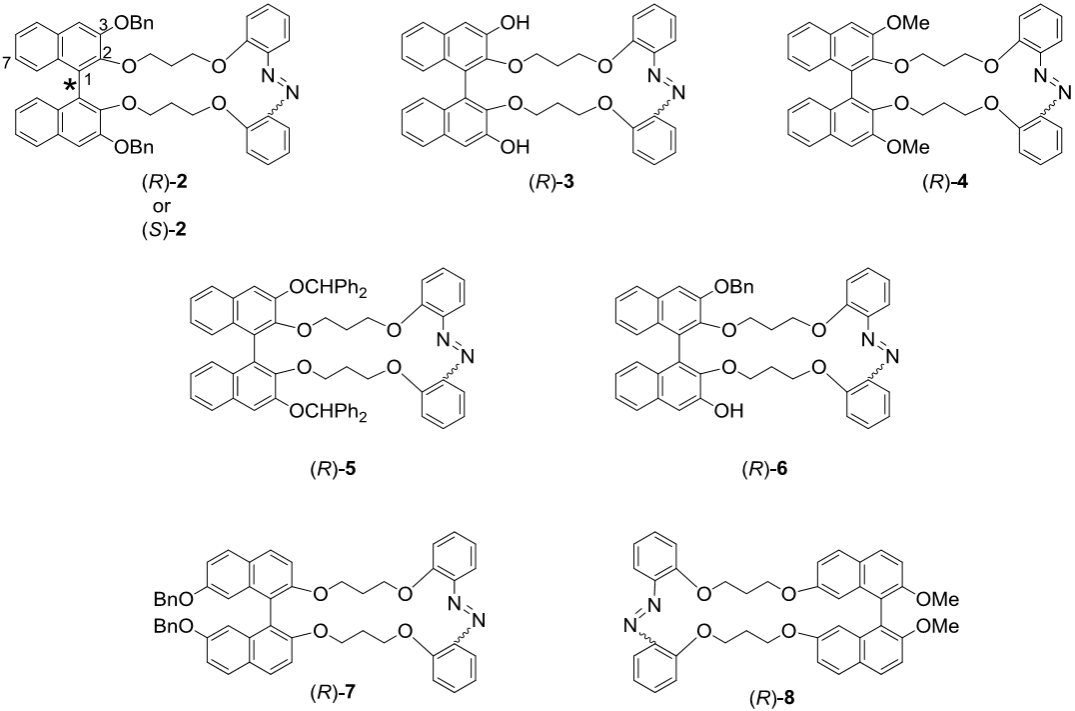
Opically active binaphthyl-azobenzene cyclic dyads **2**–**8**.

Binaphthyl-azobenzene cyclic dyads **2**–**8** used in this study are shown in Figure 3. Their binaphthyl moieties are the axially chiral parts, and the azobenzene moieties are the photochromic parts. Compounds **2**–**6** have various substituents – benzyloxy, hydroxy, methoxy, and diphenylmethoxy groups – at the 3,3’-positions of the 1,1’-binaphthyl moiety and are linked circulary to the azobenzene moiety at the 2,2’-positions. In contrast, compound **7** has dibenzyloxy groups at the 7,7’-positions, while compound **8** is substituted by 2,2’-dimethoxy groups and is linked to an azobenzene moiety through the 7,7’-positions.

### 2.2. Synthesis of 2–8

**Scheme 1:**
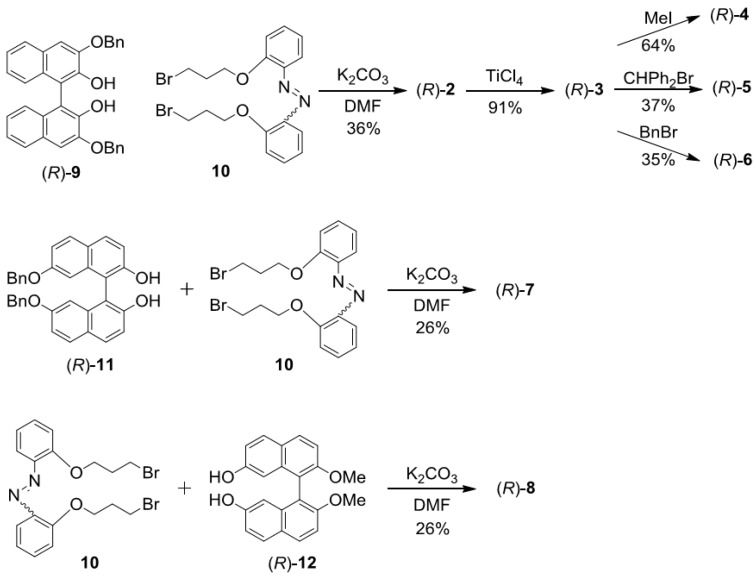
Synthetic route to (*R*)-**2**-**8**.

As shown in Scheme 1, 3,3’-disubstituted binaphthyl-azobenzene dyads (*R*)-**2**-**6** were synthesized starting from optically active (*R*)-**9** [91]. Tandem Williamson synthesis of diol (*R*)-**9** and dibromide **10** afforded cyclic compound (*R*)-**2** in 36% yield. Then debenzylation of (*R*)-**2** with titanium tetrachloride [92,93,94] for 5 min gave (*R*)-**3** in high yield (91%). Using a higher temperature, longer reaction time, or other Lewis acids (e.g. aluminum trichloride or niobium pentachloride) resulted in an extremely low yield (<5%). Additionally, generalized deprotection using palladium-activated carbon (Pd/C) and hydrogen was unsuccessful due to the concomitant reduction of the azobenzene moiety. (*R*)-**4**-**6** were prepared in moderate yields by coupling (*R*)-**3** with the appropriate halide. In the synthesis of (*R*)-**4** and -**5**, excess methyl iodide (MeI) and α-bromodiphenylmethane were used, respectively. Although in the synthesis of nonsymmetrical (*R*)-**6**, 1.1 equivalents [based on (*R*)-**3**] of benzyl bromide were used for preferential monobenzylation, mixtures of desired (*R*)-**6** (35%), unreacted (*R*)-**3** (25%), and dibenzylated (*R*)-**2** (29%) were obtained; they were easily separated by gel permeation chromatography (GPC). (*R*)-**7** and -**8** were synthesized by similar manner of synthesis of (*R*)-**2** from **10** and binol (*R*)-**11** [95,96] and -**12 **[97,98], respectively in moderate yield. Moreover, (*S*)-**2** was prepared from (*S*)-**9** and **10** using the foregoing procedure.

### 2.3. Photoswitching of Absorption, CD, and NMR Spectra

**Figure 4:**
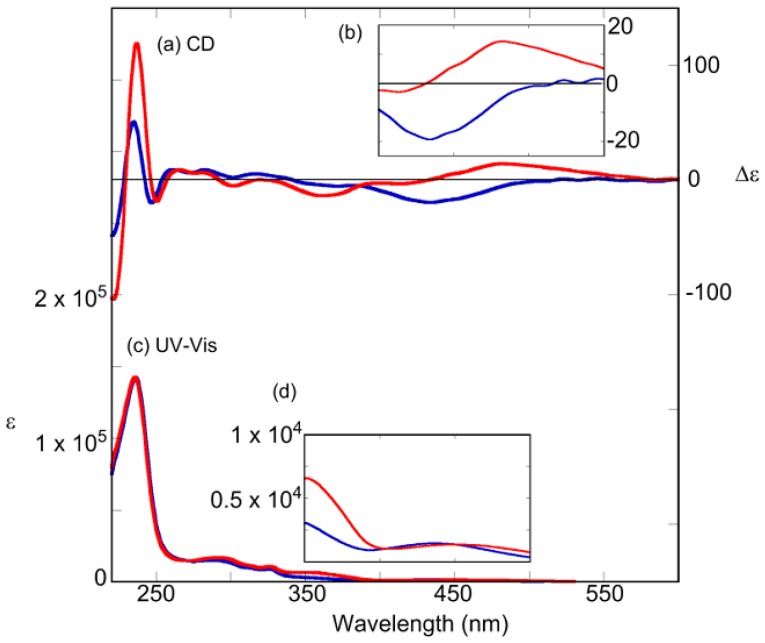
(a, b) CD spectra of (*R*)-**2**. (c, d) Absorption spectra of (*R*)-**2**. After 365-nm irradiation (blue line), after 436-nm irradiation (red line). Conditions: 1,4-dioxane (1.0 × 10^−^^5^ M), 20 °C, light path length = 10 mm, irradiation wavelength = 365 nm (10 mW/cm^2^, 100 s) and 436 nm (10 mW/cm^2^, 100 s).

**Figure 5:**
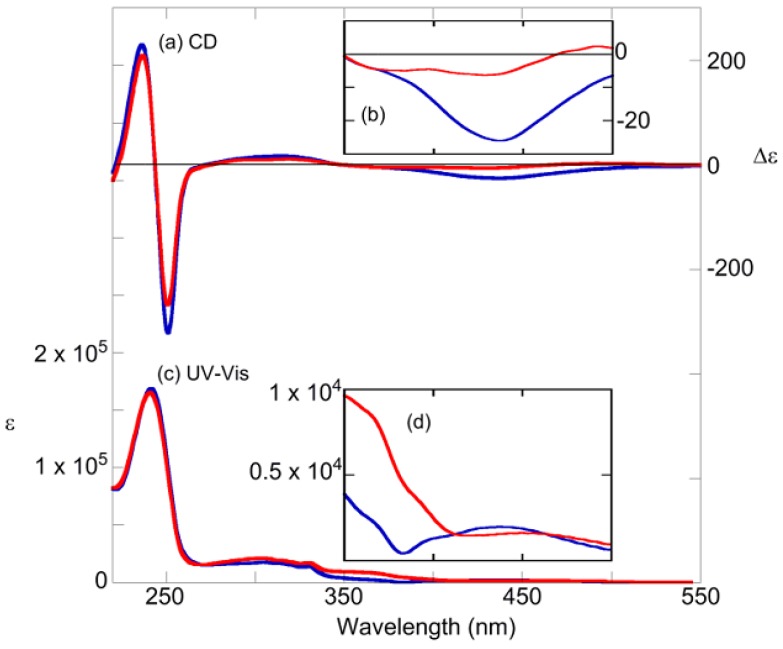
(a, b) CD spectra of (*R*)-**7**. (c, d) Absorption spectra of (*R*)-**7**. After 365-nm irradiation (blue line), after 436-nm irradiation (red line). Conditions: 1,4-dioxane (1.0 × 10^−^^5^ M), 20 °C, light path length = 10 mm, irradiation wavelength = 365 nm (10 mW/cm^2^, 100 s) and 436 nm (10 mW/cm^2^, 100 s).

The change in absorption near 360 nm, which indicated a π−π* transition of the *trans* form, confirmed the *cis-trans* isomerization of the azobenzene moiety. The absorption regions of the binaphthyl moiety were below 350 nm [95,96,97,98,99,100]. Hence, every compounds efficiently photoisomerized. As an example, Figure 4c–d and Figure 5c–d show the change in the absorption spectrum of (*R*)-**2** and (*R*)-**7** after irradiation with 365 nm or 436 nm light, respectively. Irradiation at 365 nm caused *trans*→*cis* isomerization, whereas irradiation at 436 nm caused the reverse *cis*→*trans* isomerization. Both wavelengths gave the same *cis*-*trans* isomerization rates of 0.7–0.8 in all compounds. Figure 4a–b and Figure 5a–b show the CD spectra of (*R*)-**2** and (*R*)-**7** after photoirradiation, respectively. The split CD at a short wavelength (around 250 nm), which is attributed mainly to the ^1^B_b_ transition moment of naphthalene rings, reflects the dihedral angle of two naphthalene rings of binaphthyl [101,102,103]. CD spectra of (*R*)-**2** mean the dihedral angles are varied with the compounds and the isomerism. However, further investigation on the short wavelength side is extremely difficult because the azobenzene units also absorb in this region. Meanwhile, the positive/negative region appeared on the long wavelength (400–500 nm) and absorbed only an azobenzene moiety, n-π* band, of (*R*)-**2**. Additionally, about (*R*)-**7**, the negative/flat region appeared on same wavelength. Hence, we hypothesized that their *cis*-azobenzene moieties were preferentially-twisted as either (*P*) or (*M*) along with the chiral axis of the binaphthyls. This point is discussed later in detail (Section 2.6).

**Figure 6:**
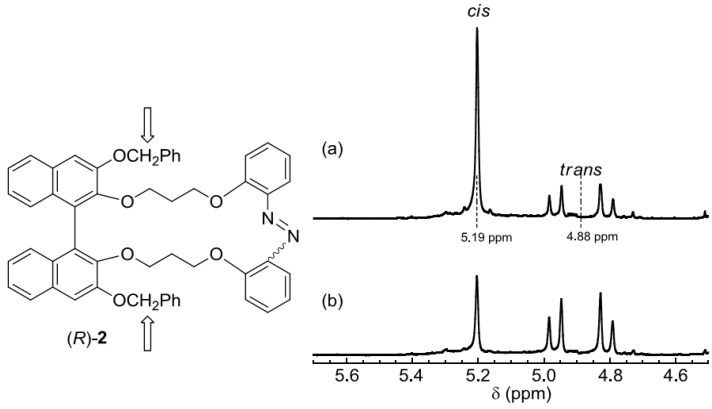
^1^H-NMR spectra of the benzyl protons of (*R*)-**2** after (a) 365 nm-irradiation, (b) 436 nm-irradiation. Conditions: CDCl_3_, 1 × 10^−3^ M, 300 MHz, 22 °C, Irradiation: 365 nm (10 mW/cm^2^, 500 s), 436 nm (10 mW/cm^2^, 500 s) in an NMR test tube (ϕ = 5 mm).

Next, the NMR spectra of the *cis*- and *trans*-forms were investigated. In compounds **2**–**8**, isomerism resulted in a pronounced change in the signals of the protons of the binaphthyl parts and their substituents far from the light-driven parts. For example, the chemical shift and signal splitting pattern of the benzyl protons far from the light-driven part of (*R*)-**2** in the ^1^H-NMR differed vastly between the *cis* and *trans* forms because the signal of (*R*)-*cis*-**2** appeared as a singlet (5.19 ppm), whereas that of (*R*)-*trans*-**2** was an AB quartet (4.88 ppm, Δν*_AB_* = 61.7 Hz, *J_AB_* = 11.2 Hz) with Δδ of 0.3 ppm (Figure 6). Like in the examples above, the 3,3’-subsituents of binaphthyls played an important role in the overall conformation of these compounds.

### 2.4. Photoswiching of Optical Rotation

**Figure 7:**
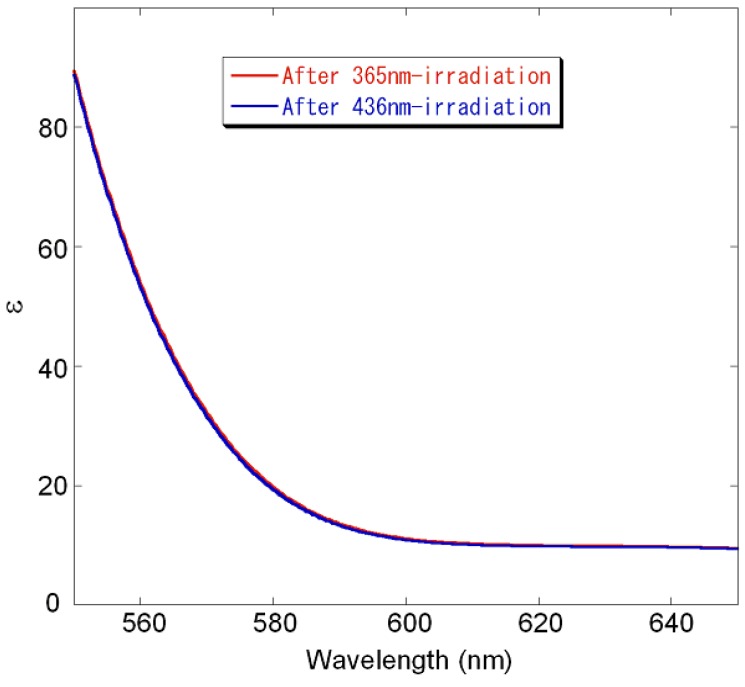
Absorption spectra of concentrated (*R*)-**2** after photo-irradiation; after 365 nm-irradiation (blue line), after 436 nm-irradiation (red line). Conditions: CHCl_3_, 0.0126 M (0.01 g/dL), light path length = 10 mm, 20 °C, Irradiation wavelength: 365 nm (10 mW/cm^2^, 500 s), 436 nm (10 mW/cm^2^, 500 s).

Isomerization also influenced the optical rotation. Table 1 shows the specific optical rotations at the sodium D-line, [α]_D_, of (*R*)-**2**-**8** after photoirradiation until the values were constant (500 seconds). Generally, [α]_D_ after 365-nm irradiation reflects the CD intensity and Cotton effect pattern at longer wavelengths. The *cis*-*trans* ratios were same as those shown in Table 1. The absolute values of [α]_D_ were lower than those of helicenes and other chiral metallic compounds [104,105,106], but were greater than general axially chiral binaphthyls. Furthermore, [α]_D_ of (*R*)-**2**, -**4**, and -**5** resulted in a sign inversion; (*R*)-**2** showed the largest change (ca. 1,000°). The absolute values of [α]_D_ of compound (*R*)-**5** remained nearly constant, but the sign was reversed. From the result of (*R*)-**6**, both 3- and 3’-substitents, which are bulkier than the hydroxy group, are necessary to realize a sufficient sign inversion. [α]_D_ of (*R*)-**7** is suited as a switch for zero-rotation/levo-rotation, although the values of (*R*)-**8** remained relatively constant despite efficient isomerization. Hence, a selection of appropriate compounds could yield any type of sign-changing pattern. Moreover, absorption at the sodium D-line (589 nm) did not occur despite analysis at high concentrations and long path-lengths (Figure 7). Thus, target compounds did not degrade during measurement of [α]_D_. Hence, a switch for large α adapted from these compounds should realize the development of nondestructive reading of memory devices [107,108,109].

**Table 1:** [α]_D_ and *cis*-*trans* ratio after photoirradiation of (*R*)-**2**-**8**.

Compound	[α]_D_ after 365 nm irradiation (deg)	[α]_D_ after 436 nm irradiation (deg)	*cis*:*trans* after 365 nm irradiation	*cis*:*trans* after 436 nm irradiation
(*R*)-**2**	−314	+642	71:29	31:69
(*R*)-**3**	−427	−490	65:35	31:69
(*R*)-**4**	−370	+248	72:28	22:78
(*R*)-**5**	−252	+278	69:31	35:65
(*R*)-**6**	−415	−285	68:32	29:71
(*R*)-**7**	−544	−7	80:20	20:80
(*R*)-**8**	+358	+282	80:20	26:74

Conditions: chloroform, c = 0.10 g/dL, 20 °C, light path length = 10 cm, irradiation wavelength = 365 nm (10 mW/cm^2^, 500 s) and 436 nm (10 mW/cm^2^, 500 s)

### 2.5. Thermodynamic Parameters of Trans to cis Isomerization Process

**Figure 8:**
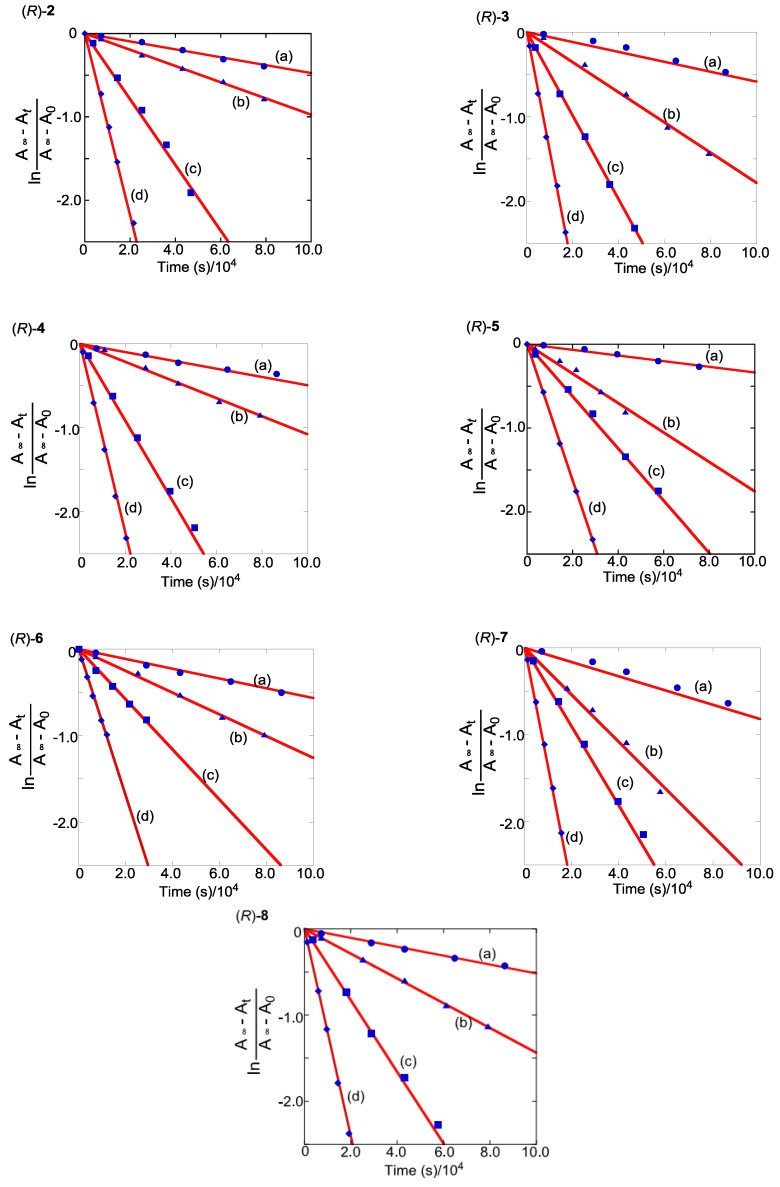
First-order plots for *cis* to *trans* thermal isomerization of (*R*)-**2-8**. (a) 35 °C, (b) 45 °C, (c) 55 °C, (d) 65 °C.Conditions: 1,4-dioxane, 1.0 × 10^−5^ M.

The thermodynamic parameters, including rate constants (*k*), enthalpy of activation (Δ*H*^‡^), entropy of activation (Δ*S*^‡^), and half-life (*t*_1/2_) at 298 K of **2**–**8** for the *cis* to *trans* thermal isomerization were measured or calculated. These parameters were determined according to Eyring equation [110] as discussed in more detail below, and were first-order reactions. For the thermal *cis*-*trans* isomerization:

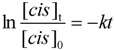
(1)
where [*cis*]*_t _*and [*cis*]_0_ are the concentrations of the *cis*-azobenzene at time *t* and time zero, respectively, and *k* is the rate constant for the thermal *cis*-*trans* isomerization. The first-order rate constant was determined by fitting the experimental data to the equation:

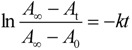
(2)
where *A_t_*, *A_0_* and *A_∞_* are the absorbance at 365 nm at time *t*, time zero and infinite time, respectively.　The first order plots according to equation (2) for the *cis*-*trans* thermal isomerization at various temperatures and resulting rate constants are shown in Figure 8 and Table 2, respectively. 

**Table 2:** Rate constants *k* (s^−1^) of *cis* to *trans* isomerization of (*R*)-**2**-**8**.

Compound	*k* at 35 °C	*k* at 45 °C	*k* at 55 °C	*k* at 65 °C
(*R*)-**2**	4.7 × 10^−6^	9.8 × 10^−^^6^	4.0 × 10^−5^	1.1 × 10^−4^
(*R*)-**3**	5.9 × 10^−6^	1.8 × 10^−5^	5.0 × 10^−5^	1.4 × 10^−4^
(*R*)-**4**	5.0 × 10^−6^	1.1 × 10^−5^	4.6 × 10^−5^	1.1 × 10^−4^
(*R*)-**5**	3.4 × 10^−6^	1.8 × 10^−5^	3.1 × 10^−5^	8.1 × 10^−^^5^
(*R*)-**6**	5.7 × 10^−6^	1.3 × 10^−5^	2.9 × 10^−5^	8.5 × 10^−5^
(*R*)-**7**	8.2 × 10^−6^	2.7 × 10^−5^	4.5 × 10^−5^	1.4 × 10^−4^
(*R*)-**8**	5.2 × 10^−6^	1.4 × 10^−5^	4.2 × 10^−5^	1.2 × 10^−4^

Conditions: 1,4-dioxane, 1.0 × 10^−^^5^ M.

Furthermore thermodynamic parameters such as enthalpy of activation (Δ*H*^‡^) and entropy of activation (Δ*S*^‡^) were determined according to the Eyring equation:

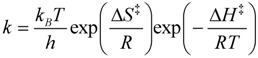
(3)
where *R*, *k_B_*, and *h* are gas constant, Boltzmann constant, and Planck’s constant, respectively. Substituting into equation (3):

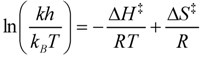
(4)

**Figure 9:**
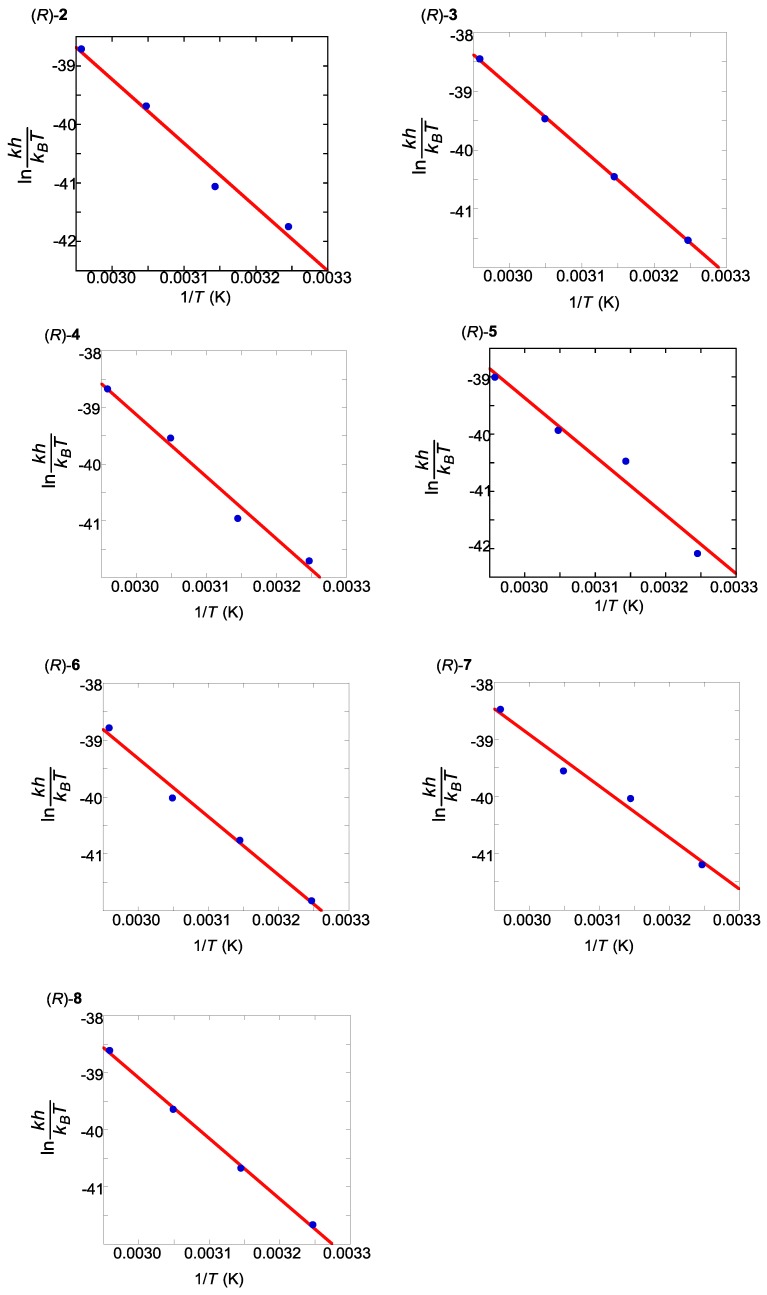
Eyring plots for *cis* to *trans* thermal isomerization of (*R*)-**2**-**8**.

Figure 9 shows Eyring plots for *cis* to *trans* thermal isomerization. The values of Δ*H*^‡^ and Δ*S*^‡^ were obtained from intersect and a slope, respectively, of the linear plot of ln(*kh*/*k_B_T*) versus 1/*T* extrapolated to *T* → ∞ (Table 3).

**Table 3:** Enthalpy of activation, entropy of activation, and half-life of (*R*)-**2**-**8**.

Compound	Δ*H*^‡^ (kcal/mol)	Δ*S*^‡^ (cal/mol·K)	*t*_1/2_ at 298 K (h)
(*R*)-**2**	22	−13	157
(*R*)-**3**	21	−14	120
(*R*)-**4**	22	−13	163
(*R*)-**5**	20	−17	148
(*R*)-**6**	18	−25	101
(*R*)-**7**	18	−23	61
(*R*)-**8**	21	−15	128

Conditions: 1,4-dioxane, 1.0 × 10^−5^ M.

Both Δ*H*^‡^ and Δ*S*^‡^ derived from the rate constants varied slightly for each compound. Although the isomer ratios differ slightly, it is more than probable that the isomerization mechanism is the same for all compounds. Furthermore, most of these compounds had the half-lives at 298 K longer than 100 h, which is extraordinary for azobenzene derivatives. These long lives provide practical advantages for future application.

### 2.6. Helical Chirality of cis-Azobenzenes

**Figure 10:**
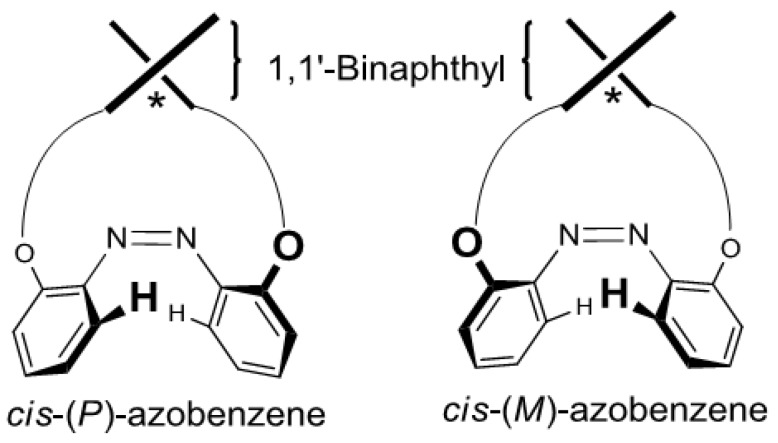
Structures of *cis*-(*P*)-azobenzene and *cis*-(*M*)-azobenzenelinked to a 1,1’-binaphthyl.

To confirm the hypothesis that the axial chirality of binaphthyl induces the helical chirality of *cis*-azobenzene (Figure 10), we calculated the optimized geometries and corresponding CD spectra of two diastereomers, the (*P*)- and (*M*)-forms at the azobenzene moiety. 

**Figure 11:**
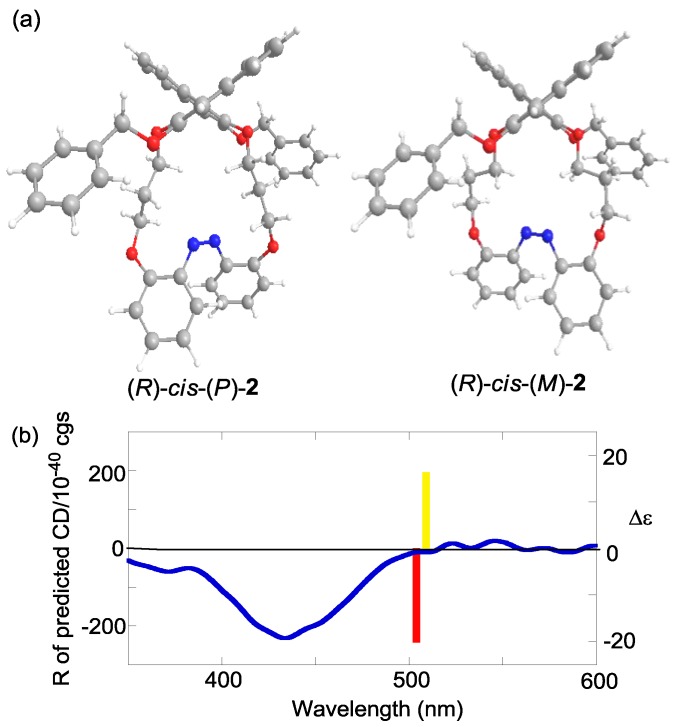
(a) Optimized structures of (*R*)-*cis*-(*P*)-**2** and (*R*)-*cis*-(*M*)-**2** obtained by the DFT calculations at the B3LYP/6-31G(d) level under *C_2_* symmetry. (b) CD calculated by the TD-DFT method with the B3LYP/6-31G(d) level of (*R*)-*cis*-(*P*)-**2** (red bar), (*R*)-*cis*-(*M*)-**2** (yellow bar), and experimental CD of (*R*)-**2** after 365-nm irradiation (blue line, 1 × 10^−5^ M in 1,4-dioxane, 20 °C).

Figure 11a shows the optimized structures of (*R*)-*cis*-(*P*)- and (*R*)-*cis*-(*M*)-**2** obtained by the DFT calculations at the B3LYP/6-31G(d) level [101]. Figure 11b shows the CD in the “azobenzene region” (350–600 nm) predicted using these optimized structures by TD-DFT methods at the B3LYP/6-31G(d) level. These results indicated that the (*R*)-*cis*-(*P*)-**2** exhibited a negative Cotton effect pattern and (*R*)-*cis*-(*M*)-**2** exhibited a positive Cotton effect pattern. 

**Figure 12:**
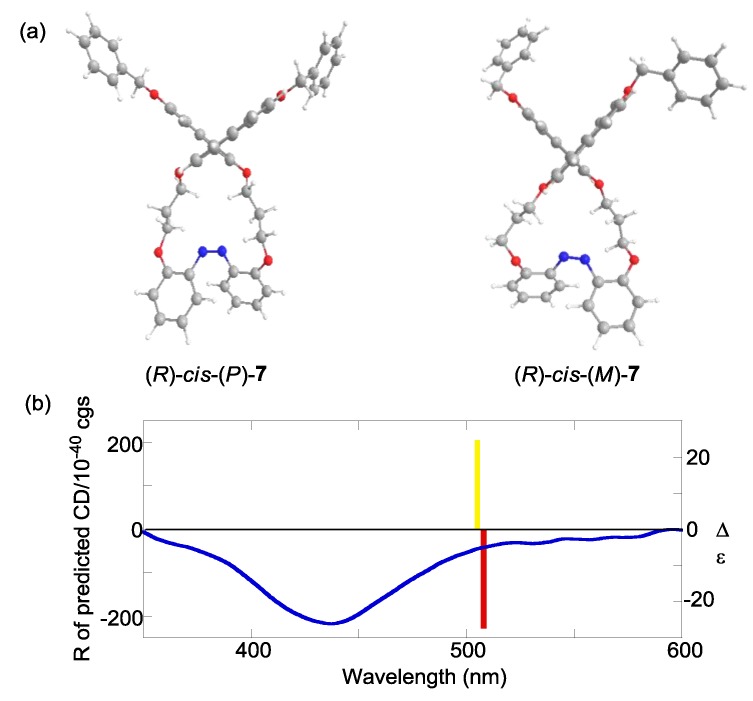
(a) Optimized structures of (*R*)-*cis*-(*P*)-**7** and (*R*)-*cis*-(*M*)-**7** obtained by the DFT calculations at the B3LYP/6-31G(d) level. (b) CD calculated by the TD-DFT method with the B3LYP/6-31G(d) level of (*R*)-*cis*-(*P*)-**7** (red bar), (*R*)-*cis*-(*M*)-**7** (yellow bar), and experimental CD of (*R*)-**7** after 365-nm irradiation (blue line, 1 × 10^−5^ M in 1,4-dioxane, 20 °C).

**Figure 13:**
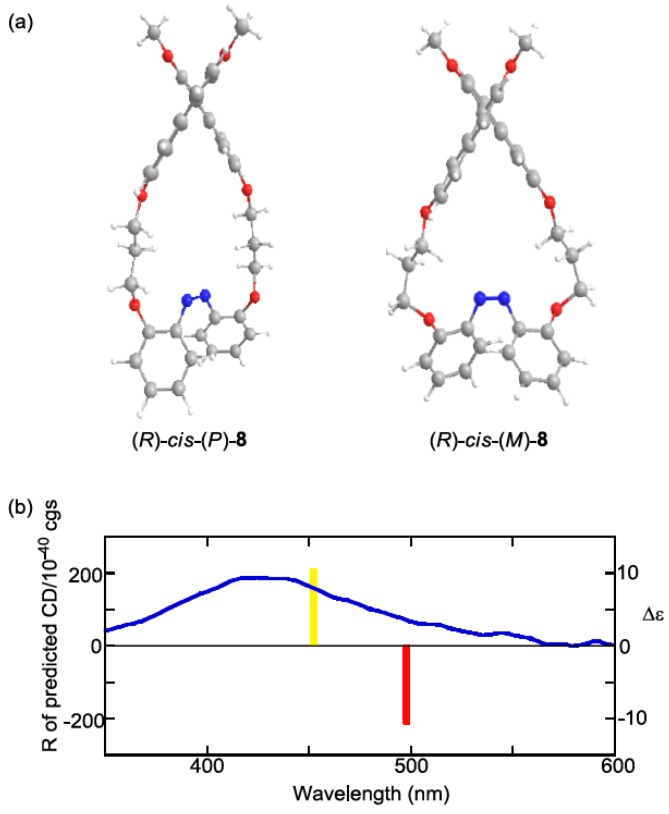
(a) Optimized structures of (*R*)-*cis*-(*P*)-**8** and (*R*)-*cis*-(*M*)-**8** obtained by the DFT calculations at the B3LYP/6-31G(d) level under *C_2_* symmetry. (b) CD calculated by the TD-DFT method with the B3LYP/6-31G(d) level of (*R*)-*cis*-(*P*)-**8** (red bar), (*R*)-*cis*-(*M*)-**7** (yellow bar), and experimental CD of (*R*)-**8** after 365-nm irradiation (blue line, 1 × 10^−5^ M in 1,4-dioxane, 20 °C).

**Figure 14:**
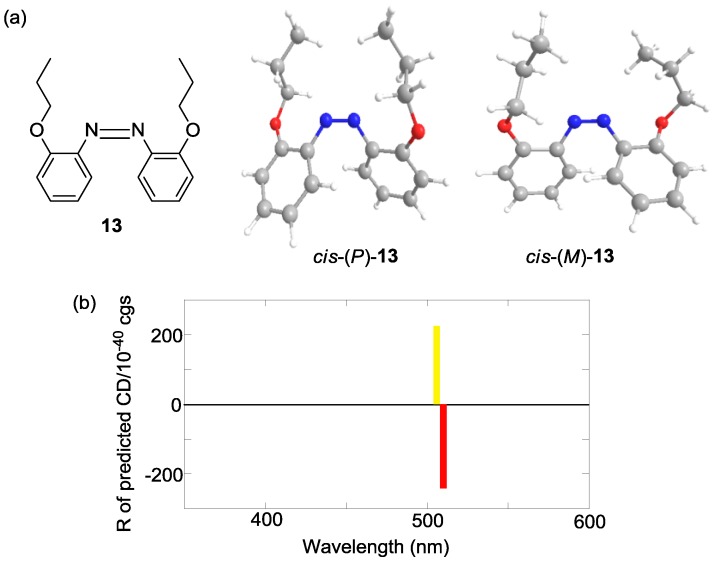
(a) Structures of *cis*-(*P*)-**13** and *cis*-(*M*)-**13**. (b) CD calculated by the TD-DFT method with the B3LYP/6-31G(d) level of *cis*-(*P*)-**13** (red bar), *cis*-(*M*)-**13** (yellow bar).

The experimental CD indicated that the preferential configuration of (*R*)-*cis*-**2 **was the (*P*)-form. Using the same method, (*R*)-*cis*-**7** was also determined to assume (*P*)-configuration (Figure 12). In contrast, (*R*)-*cis*-**8** was determined to assume (*M*)-configuration (Figure 13). Additionally, the CDs of just the azobenzene moieties in *cis*-(*P*)- and *cis*-(*M*)-**13** were calculated (Figure 14). Similar to (*R*)-*cis*-**2** -**7**, and -**8**, *cis*-(*P*)-**13** (*cis*-(*M*)-**13**) exhibited a negative (positive) Cotton effect. Therefore, the CD patterns described herein are derived from the innate CD of azobenzene itself and not from binaphthyl-related induced CD [112,113]. Most of the optimized structures were obtained by a calculation under *C_2_* symmetry. However, the obtained structure of (*R*)-*cis*-(*M*)-**7 **calculated under *C_2_* symmetry had an imaginary frequency. Hence, the symmetry was ignored during the optimization of (*R*)-*cis*-(*M*)-**7**. Additionally, some of the obtained structures using M06/6-31G(d) [114] were unacceptable as it was based on an imaginary frequency.

**Figure 15:**
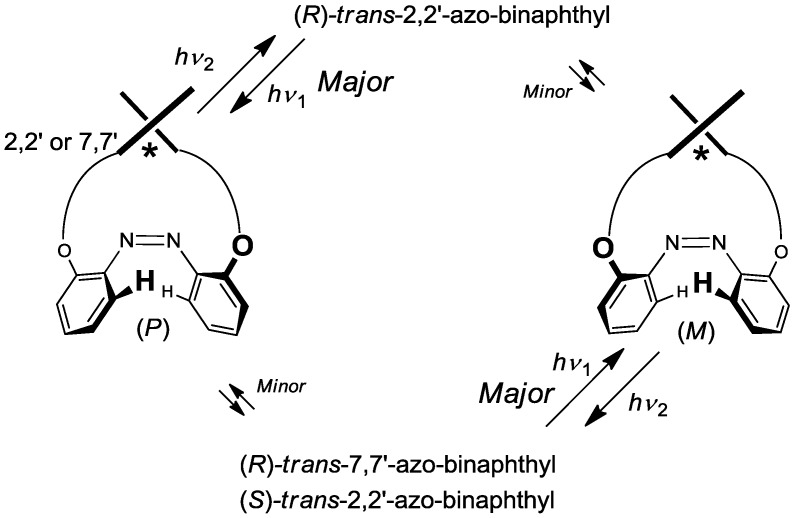
Twisting pattern of *cis*-azobenzenes induced by axial chirality of binaphthyls.

Table 4, which shows the data regarding the asymmetry of the compounds (*R*)-**2**-**8**, (*S*)-**2**, and **13**, illustrates three points regarding chirality: (1) (*R*)-2,2’-azobenzene-linked-binaphthyls induced (*P*)-azobenzene, (2) (*S*)-2,2’-analogues induced (*M*)-azobenzene, and (3) 7,7’-analogues acted as pseudo-enantiomers of 2,2’-analogues. A systematic and simple induction of asymmetrical *cis*-azobenzenes (Figure 15) was structured using common binaphthyl skeletons. These conformational results should assist in future studies on asymmetric azobenzenes.

**Table 4:** Sign of experimental and calculated CD, and helical chirality of the *cis*-azobenzene moiety in **2**–**8** and **13**.

Compound	Sign of Δε^[a][b]^			
	Experimental	Calculated *cis*-(*P*)-form	Calculated *cis*-(*M*)-form	Chirality of *cis*-azobenzene^[c]^
(*R*)-**2**	–	–	+	(*P*)
(*R*)-**3**	–	–	+	(*P*)
(*R*)-**4**	–	–	+	(*P*)
(*R*)-**5**	–	–	+	(*P*)
(*R*)-**6**	–	–	+	(*P*)
(*R*)-**7**	–	–	+	(*P*)
(*R*)-**8**	+	–	+	(*M*)
(*S*)-**2**	+	–	+	(*M*)
**1** **3**		–	+	

[a] Sign of Δε at 350–600 nm; [b] Calculated using the TD-DFT method with B3LYP/6-31G(d); [c] These chiralities were determined by comparing the signs between the experimental and computed CD.

## 3. Conclusions

Several axially chiral binaphthyl-azobenzene dyads with substituents were synthesized to examine their reversible photoisomerization and subsequent changes in optical properties. The chiroptical properties of the dyads, CD and optical rotation, changed dramatically. It is notable that reversible positive-negative and zero-positive (zero-negative) chiroptical signals were detected. Moreover, intramolecular chiral transfer from the chiral axis to the helix of the *cis*-azobenzene moiety was studied empirically and computationally. 2,2’-Linked-(*R*)-binaphthyl was found to induce *cis*-(*P*)-azobenzene, whereas the symmetrical 7,7’-linked-(*R*)-binaphthyl was found to induce *cis*-(*M*)-azobenzene without exception. These results may be useful in designing chiroptical switches as well as in the study of azobenene helicity. Additionally, this work provides a novel use for axially chiral binaphthyls. We are currently studying the development, conformation, and application of specific binaphthyl-azobenzene systems with interesting functions.

## References

[1] Ogoshi T., Shiga R., Yamagishi T., Nakamoto Y. (2011). Planar-chiral pillar[5]arene: Chiral switches induced by multiexternal stimulus of temperature, solvents, and addition of achiral guest molecule. J. Org. Chem..

[2] Liu W., Cao D., Peng J., Zhang H., Meier H. (2010). A dendrimer chiroptical switch based on the reversible intramolecular photoreaction of anthracene and benzene rings. Chem. Asian J..

[3] Zheng Y., Cui J., Zheng J., Wan X. (2010). Near-infrared electrochromic and chiroptical switching polymers: Synthesis and characterization of helical poly(*N*-propargylamides) carrying anthraquinone imide moieties in side chains. J. Mater. Chem..

[4] Li D., Wang Z.Y., Ma D. (2009). Electrically-controlled near-infrared chiroptical switching of enantiomeric dinuclear ruthenium complexes. Chem. Commun..

[5] Deng J., Zhou C., Chen C., Song N., Su Z. (2008). Synthesis and redox-driven chiroptically switching properties of viologen-containing optically active polymer with main-chain axial chirality. Macromolecules.

[6] Deng J., Song N., Zhou Q., Su Z. (2007). Electrically-driven chiroptical switches based on axially dissymmetric 1,1’-binaphthyl and electrochromic viologens: Synthesis and optical properties. Org. Lett..

[7] Rajakumar P., Selvam S. (2007). Synthesis, complexation, and photoisomerization studies on some chiral monocyclic stilbenophanes and bis-cyclophanes. Tetrahedron.

[8] van Delden R.A., Mecca T., Rosini C., Feringa B.L. (2004). A chiroptical molecular switch with distinct chiral and photochromic entities and its application in optical switching of a cholesteric liquid crystal. Chem. Eur. J..

[9] Feringa B.L. (2001). In control of motion: From molecular switches to molecular motors. Acc. Chem. Res..

[10] Cornelissen J.J.L.M., Rowan A.E., Nolte R.J.M., Sommerdijk N.A.J.M. (2001). Chiral architectures from macromolecular building blocks. Chem. Rev..

[11] Feringa B.L., van Delden R.A., Koumura N., Geertsema E.M. (2000). Chiroptical molecular switches. Chem. Rev..

[12] Brunel J.M. (2005). BINOL: A versatile chiral reagent. Chem. Rev..

[13] Chen Y., Yekta S., Yudin A. K. (2003). Modified BINOL ligands in asymmetric catalysis. Chem. Rev..

[14] Berthod M., Mignani G., Woodward G., Lemaire M. (2005). Modified BINAP: The How and the Why. Chem. Rev..

[15] Miyashita A., Yasuda A., Takaya H., Toriumi K., Ito T., Souchi T., Noyori R. (1980). Synthesis of 2,2’-bis(dipheny1phosphino)-1,l’-binaphthyl (BINAP), an atropisomeric chiral bis(triaryl)-phosphine, and its use in the rhodium(I)-catalyzed asymmetric hydrogenation of a (acylamino)acrylic acids. J. Am. Chem. Soc..

[16] Yukawa T., Seelig B., Xu Y., Morimoto H., Matsunaga S., Berkessel A., Shibasaki M. (2010). Catalytic asymmetric aza-Morita−Baylis−Hillman reaction of methyl acrylate: Role of a bifunctional La(O-*i*Pr)_3_/linked-BINOL complex. J. Am. Chem. Soc..

[17] Mirri G., Bull S.D., Horton P.N., James T.D., Male L., Tucker J.H.R. (2010). Electrochemical method for the determination of enantiomeric excess of binol using redox-active boronic acids as chiral sensors. J. Am. Chem. Soc..

[18] Pignataro L., Carboni S., Civera M., Colombo R., Piarulli U., Gennari C. (2010). PhthalaPhos: Chiral supramolecular ligands for enantioselective rhodium-catalyzed hydrogenation reactions. Angew. Chem. Int. Ed..

[19] Li N., Chen X.-H., Zhou S.-M., Luo S.-W., Song J., Ren L., Gong L.-Z. (2010). Asymmetric Amplification in Phosphoric Acid Catalyzed Reactions. Angew. Chem. Int. Ed..

[20] Hou Z., Wang J., He P., Wang J., Qin B., Liu X., Lin L., Feng X. (2010). Highly enantioselective insertion of carbenoids into N-H bonds catalyzed by copper(I) complexes of binol derivatives. Angew. Chem. Int. Ed..

[21] Shirakura M., Suginome M. (2010). Nickel-catalyzed asymmetric addition of alkyne C-H bonds across1,3-dienes using taddol-based chiral phosphoramidite ligands. Angew. Chem. Int. Ed..

[22] Hatano M., Moriyama K., Maki T., Ishihara K. (2010). Which is the actual catalyst: Chiral phosphoric acid or chiral calcium phosphate?. Angew. Chem. Int. Ed..

[23] Teichert J.F., Feringa B.L. (2010). Phosphoramidites: Privileged ligands in asymmetric catalysis. Angew. Chem. Int. Ed..

[24] Aikawa K., Hioki Y., Mikami K. (2010). Highly enantioselective alkynylation of trifluoropyruvate with alkynylsilanes catalyzed by the BINAP-Pd complex: Access to α-trifluoromethyl-substituted tertiary alcohols. Org. Lett..

[25] Arai N., Akashi M., Sugizaki S., Ooka H., Inoue T., Ohkuma T. (2010). Asymmetric hydrogenation of bicyclic ketones catalyzed by BINAP/IPHAN-Ru(II) complex. Org. Lett..

[26] Fraile J.M., Garcia J.I., Mayoral J.A. (2009). Noncovalent Immobilization of Enantioselective Catalysts. Chem. Rev..

[27] Yamamoto H., Abel J.P. (2008). Catalytic enantioselective pudovik reaction of aldehydes and aldimines with tethered bis(8-quinolinato) (TBOx) aluminum complex. J. Am. Chem. Soc..

[28] Rabalakos C., Wulff W.D. (2008). Enantioselective organocatalytic direct Michael addition of nitroalkanes to nitroalkenes promoted by a unique bifunctional DMAP-thiourea. J. Am. Chem. Soc..

[29] Chen X., Huang Z., Chen S.-Y., Li K., Yu X.-Q., Pu L. (2010). Enantioselective gel collapsing: A new means of visual chiral sensing. J. Am. Chem. Soc..

[30] Liu H.-L., Zhu H.-P., Hou X.-L., Lin Pu L. (2010). Highly enantioselective fluorescent recognition of serine and other amino acid derivatives. Org. Lett..

[31] Liu H.-L., Peng Q., Wu Y.-D., Chen D., Hou X.-L., Sabat M., Pu L. (2010). Highly enantioselective recognition of structurally diverse α-hydroxycarboxylic acids using a fluorescent sensor. Angew. Chem. Int. Ed..

[32] Sambasivan S., Kim D., Ahn K.H. (2010). Chiral discrimination of α-amino acids with a *C*_2_-symmetric homoditopic receptor. Chem. Commun..

[33] Yang L., Qin S., Su X., Yang F., You J., Hu C., Xie R., Lan J. (2010). 1,1’-Binaphthyl-based imidazolium chemosensors for highly selective recognition of tryptophan in aqueous solutions. Org. Biomol. Chem..

[34] Ema T., Hamada K., Sugita K., Nagata Y., Sakai T., Ohnishi A. (2010). Synthesis and evaluation of chiral selectors with multiple hydrogen-bonding sites in the macrocyclic cavities. J. Org. Chem..

[35] Nandhakumar R., Ryu J., Park H., Tang L., Choi S., Kim K.M. (2008). Effects of ring substituents on enantioselective recognition of amino alcohols and acids in uryl-based binol receptors. Tetrahedron.

[36] Ema T., Tanida D., Sugita K., Sakai T., Miyazawa K., Ohnishi A. (2008). Chiral selector with multiple hydrogen-bonding sites in a macrocyclic cavity. Org. Lett..

[37] Ema T., Tanida D., Hamada K., Sakai T. (2008). Tuning the chiral cavity of macrocyclic receptor for chiralrecognition and discrimination. J. Org. Chem..

[38] Park H., Nandhakumar R., Hong J., Ham S., Chin J., Kim K. M. (2008). Stereoconversion of amino acids and peptides in uryl-pendant binol schiff bases. Chem. Eur. J..

[39] Wang Q., Chen X., Tao L., Wang L., Xiao D., Yu X.-Q., Pu L. (2007). Enantioselective fluorescent recognition of amino alcohols by a chiral tetrahydroxyl 1,1’-binaphthyl compound. J. Org. Chem..

[40] Bříza T., Kejík Z., Vašek P., Králová J., Martásek P., Císařová I., Král V. (2005). Chromophoric binaphthylderivatives. Org. Lett..

[41] Tsubaki K., Tanaka H., Morikawa H., Fuji K. (2003). Synthesis and recognition of amino acids by binaphthyl-crown receptors. Tetrahedron.

[42] Mathews M., Zola R.S., Hurley S., Yang D.-K., White T.J., Bunning T.J., Li Q. (2010). Light-driven reversible handedness inversion in self-organized helical superstructures. J. Am. Chem. Soc..

[43] Han Y., Pacheco K., Bastiaansen C.W.M., Broer D.J., Sijbesma R.P. (2010). Optical monitoring of gases with cholesteric liquid crystals. J. Am. Chem. Soc..

[44] Akagi K. (2009). Helical polyacetylene: Asymmetric polymerization in a chiral liquid-crystal field. Chem. Rev..

[45] Yoshizawa A., Kogawa Y., Kobayashi K., Takanishi Y., Yamamoto J. (2009). A binaphthyl derivative with a wide temperature range of a blue phase. J. Mater. Chem..

[46] Goh M., Kyotani M., Akagi K. (2007). Highly twisted helical polyacetylene with morphology free from the bundle of fibrils synthesized in chiral nematic liquid crystal reaction field. J. Am. Chem. Soc..

[47] Yoshizawa A., Kobayashi K., Sato M. (2007). Host-guest effect on chirality transfer from a binaphthyl derivative to a host nematic liquid crystal. Chem. Commun..

[48] Li Q., Green L., Venkataraman N., Shiyanovskaya I., Khan A., Urbas A., Doane J.W. (2007). Reversible photoswitchable axially chiral dopants with high helical twisting power. J. Am. Chem. Soc..

[49] Eelkema R., Feringa B.L. (2006). Amplification of chirality in liquid crystals. Org. Biomol. Chem..

[50] Pieraccini S., Ferrarini A., Fuji K., Gottarelli G., Lena S., Tsubaki K., Spada G.P. (2006). Homochiral helices of oligonaphthalenes inducing opposite-handed cholesteric phases. Chem. Eur. J..

[51] Akagi K., Guo S., Mori T., Goh M., Piao G., Kyotan M. (2005). Synthesis of helical polyacetylene in chiral nematic liquid crystals using crown ether type binaphthyl derivatives as chiral dopants. J. Am. Chem. Soc..

[52] Holzwarth R., Bartsch R., Cherkaoui Z., Solladié G. (2004). New 2,2'-substituted 4,4'-dimethoxy-6,6'-dimethyl[1,1'-biphenyls], inducing a strong helical twisting power in liquid crystals. Chem. Eur. J..

[53] Carlo R., Piero S.G., Gloria P., Stefano M., Simone S. (1997). Conformational analysis of some trans-4,5-Diaryl-1,3-dioxolanes by CD spectroscopy and induction of cholesteric mesophases in nematic solvents: A correlation between twisting power and structure of the dopant. J. Am. Chem. Soc..

[54] Gottarelli G., Spada G.P. (1986). Induction of the cholesteric mesophase in nematic liquid crystals: Correlation between the conformation of open-chain chiral 1,l’-binaphthyls and their twisting powers. J. Org. Chem..

[55] Gottarelli G., Hibert M., Samori B., Solladie G., Spada G.P., Zimmermann R. (1983). Induction of the cholesteric mesophase in nematic liquid crystals: Mechanism and application to the determination of bridged biaryl configurations. J. Am. Chem. Soc..

[56] Reimann S., Urakawa A., Baiker A. (2010). BINAP adsorption on palladium: A combined infrared spectroscopy and theoretical study. J. Phys. Chem. C.

[57] Nishizaka M., Mori T., Inoue Y. (2010). Experimental and theoretical studies on the chiroptical properties of donor-acceptor binaphthyls. Effects of dynamic conformer population on circular dichroism. J. Phys. Chem. Lett..

[58] Bunzen J., Bruhn T., Bringmann G., Lűtzen A. (2009). Synthesis and helicate formation of a new family of BINOL-based bis(bipyridine) ligands. J. Am. Chem. Soc..

[59] Sahnoun R., Koseki S., Fujimura Y. (2006). Density functional theoretical study on enantiomerization of 2,2'-biphenol. J. Phys. Chem. A.

[60] Albrow V., Biswas K., Crane A., Chaplin N., Easun T., Gladiali S., Lygo B., Woodward S. (2003). Synthesis of an octahydro-1,1’-binaphthyl thioether ligand and comparison with unhydrogenated binaphthyl analogues. Tetrahedron Asymmetry.

[61] Setnička V., Urbanová M., Bouř P., Král V., Volka K. (2001). Vibrational circular dichroism of 1,1’-binaphthyl derivatives: Experimental and theoretical study. J. Phys. Chem. A.

[62] Kranz M., Clark T., Schleyer P.v.R. (1993). Rotational barriers of 1,1'-binaphthyls: A computational study. J. Org. Chem..

[63] Dube H., Ams M.R., Rebek J. (2010). Supramolecular control of fluorescence through reversible encapsulation. J. Am. Chem. Soc..

[64] Han J., Yan D., Shi W., Ma J., Yan H., Wei M., Evans D.G., Duan X. (2010). Layer-by-layer ultrathin films of azobenzene-containing polymer/layered double hydroxides with reversible photoresponsive behavior. J. Phys. Chem. B.

[65] Liu M., Yan X., Hu M., Chen X., Zhang M., Zheng B., Hu X., Shao S., Huang F. (2010). Photoresponsive host-guest systems based on a new azobenzene-containing crytpand. Org. Lett..

[66] Oka Y., Tamaoki N. (2010). Structure of silver(I) complex prepared from azobenzenonaphthalenophane, photochemical coordination change of silver(I) and silver(I)-induced acceleration of *Z*-*E* thermal isomerization of azobenzene unit. Inorg. Chem..

[67] Kawamoto M., Sassa T., Wada T. (2010). Photoinduced control over the self-organized orientation of amorphous molecular materials using polarized light. J. Phys. Chem. B.

[68] Chen W.-C., Lee Y.-W., Chen C.-T. (2010). Diastereoselective, synergistic dual-mode optical switch with integrated chirochromic helicene and photochromic bis-azobenzene moieties. Org. Lett..

[69] Basheer M.C., Oka Y., Mathews M., Tamaoki N. (2010). A light-controlled molecular brake with complete ON-OFF rotation. Chem. Eur. J..

[70] Kawamoto M., Shiga N., Takaishi K., Yamashita T. (2010). Non-destructive erasable molecular switches and memory using light-driven twisting motions. Chem. Commun..

[71] Sadovski O., Beharry A.A., Zhang F., Woolley G.A. (2009). Spectral tuning of azobenzene photoswitches for biological applications. Angew. Chem. Int. Ed..

[72] Zou G., Jiang H., Kohn H., Manaka T., Iwamoto M. (2009). Control and modulation of chirality for azobenzene-substituted polydiacetylene LB films with circularly polarized light. Chem. Commun..

[73] Siewertsen R., Neumann H., Buchheim-Stehn B., Herges R., Näther C., Renth F., Temps F. (2009). Highly efficient reversible *Z-E* photoisomerization of a bridged azobenzene with visible light through resolved S1(nπ*) absorption bands. J. Am. Chem. Soc..

[74] Uno S., Dohno C., Bittermann H., Malinovskii V.L., Häner R., Nakatani K. (2009). A light-driven supramolecular optical switch. Angew. Chem. Int. Ed..

[75] Chen J., Serizawa T., Komiyama M. (2009). Peptides recognize photoresponsive targets. Angew. Chem. Int. Ed..

[76] Puntoriero F., Bergamini G., Ceroni P., Balzania V., Vögtle F. (2008). A fluorescent guest encapsulated by a photoreactive azobenzene dendrimer. New J. Chem..

[77] Tamaoki N., Mathews M. (2008). Planar chiral azobenzenophanes as chiroptic switches for photon mode reversible reflection color control in induced chiral nematic liquid crystals. J. Am. Chem. Soc..

[78] King E.D., Tao P., Sanan T.T., Hadad C.M., Parquetted J.R. (2008). Photomodulated chiral induction in helical azobenzene oligomers. Org. Lett..

[79] Kay E.R., Leigh D.A., Zerbetto F. (2007). Synthetic molecular motors and mechanical machines. Angew. Chem. Int. Ed..

[80] Vera F., Tejedor R.M., Romero P., Barberá J., Ros M.B., Serrano J.L., Sierra T. (2007). Light-driven supramolecular chirality in propeller-like hydrogen-bonded complexes that show columnar mesomorphism. Angew. Chem. Int. Ed..

[81] Alam M.Z., Yoshioka T., Ogata T., Nonaka T., Kurihara S. (2007). Influence of helical twisting poweron the photoswitching behavior of chiral azobenzene compounds: Applications to high-performance switching devices. Chem. Eur. J..

[82] Choi S.-W., Kawauchi S., Ha N.Y., Takezoe H. (2007). Photoinduced chirality in azobenzene-containing polymer systems. Phys. Chem. Chem. Phys..

[83] Muraoka T., Kinbara K., Aida T. (2006). Mechanical twisting of a guest by a photoresponsive host. Nature.

[84] Jousselme B., Blanchard P., Allain M., Levillain E., Dias M., Roncali J. (2006). Structural control of the electronic properties of photodynamic azobenzene-derivatized π-conjugated oligothiophenes. J. Phys. Chem. A.

[85] Balzani V., Venturi M., Credi A. (2003). Molecular Devices and Machines.

[86] Feringa B.L. (2001). Molecular Switches.

[87] Kawamoto M., Aoki T., Wada T. (2007). Light-driven twisting behaviour of chiral cyclic compounds. Chem. Commun..

[88] Takaishi K., Kawamoto M., Tsubaki K., Wada T. (2009). Photoswitching of dextro/levo rotation with axially chiral binaphthyls linked to an azobenzene. J. Org. Chem..

[89] Takaishi K., Kawamoto M., Tsubaki K., Furuyama T., Muranaka A., Uchiyama M. (2011). Helical chirality of azobenzenes induced by an intramolecular chiral axis and potential as chiroptical switches. Chem. Eur. J..

[90] Haberhauer G., Kallweit C. (2010). A bridged azobenzene derivative as a reversible, light-induced chirality switch. Angew. Chem. Int. Ed..

[91] Tsubaki K., Morikawa H., Tanaka H., Fuji K. (2003). Convenient synthesis and efficient resolution of 3,3'-bis(benzyloxy)-1,1’-binaphthalene-2,2’-diol. Tetrahedron Asymmetry.

[92] Chang C.-F., Yang L.-Y., Chang S.-W., Fang Y.-T., Lee Y.-J. (2008). Total synthesis of demethylwedelolactone and wedelolactone by Cu-mediated/Pd(0)-catalysis and oxidative-cyclization. Tetrahedron.

[93] Surivet J.-P., Vatèle J.-M. (1998). First total synthesis of (-)-8-epi-9-deoxygoniopypyrone. Tetrahedron Lett..

[94] Hori H., Nishida Y., Ohrui H., Meguro H. (1989). Regioselective de-*O*-benzylation with lewis acids. J. Org. Chem..

[95] Bandin M., Casolari S., Cozzi P.G., Proni G., Schmohel E., Spada G.P., Tagliavini E., Umani-Ronchi A. (2000). Synthesis and characterization of new enantiopure 7,7’-disubstituted 2,2’-dihydroxy-1,1’-binaphthyls: Useful ligands for the asymmetric allylation reaction of aldehydes. Eur. J. Org. Chem..

[96] Diederich F., Hester M.R., Uyeki M.A. (1988). Auf 2,2’,7,7’-Tetrahydroxy-1,1’-binaphthyl basierende neuartige chirale mono- und ditope cyclophanartige wirtverbindungen mit einer unpolaren bindungsstelle. Angew. Chem..

[97] Horiuchi T., Ohta T., Stephan M., Takaya H. (1994). Synthesis of (*R*)- and (*S*)-7,7’-bis(diphenylphosphino)-2,2’-dimethoxy-l,l’-binaphthyl, a new axially dissymmetric bis(triarylphosphine). Tetrahedron Asymmetry.

[98] Reeder J., Castro P.P., Knobler C.B. (1994). Chiral recognition of cinchona alkaloids at the minor and major grooves of 1,l’-binaphthyl receptors. J. Org. Chem..

[99] Takaishi K., Sue D., Kuwahara S., Harada N., Kawabata T., Tsubaki K. (2009). Synthesis and properties of *S*,*R*-alternating octinaphthalenes. Tetrahedron.

[100] Sue D., Takaishi K., Harada T., Kuroda R., Kawabata T., Tsubaki K. (2009). Synthesis of chiral dotriacontanaphthalenes: How many naphthalene units are we able to elaborately connect?. J. Org. Chem..

[101] Bari L.D., Pescitelli G., Marchetti F., Salvadori P. (2000). Anomalous CD/UV exciton splitting of a binaphthyl derivative: The case of 2,2’-diiodo-1,1’-binaphthalene. J. Am. Chem. Soc..

[102] Harada N., Nakanishi K. (1972). The exciton chirality method and its application to configurational and conformational studies of natural products. Acc. Chem. Res..

[103] Jaffé H.H., Orchin M. (1962). Theory and Applications of Ultraviolet Spectroscopy.

[104] Graule S., Rudolph M., Vanthuyne N., Autschbach J., Roussel C., Crassous J., Réau R. (2009). Metal-bis(helicene) assemblies incorporating π-conjugated phosphole-azahelicene ligands: Impacting chiroptical properties by metal variation. J. Am. Chem. Soc..

[105] Rzepa H.S. (2009). The chiro-optical properties of a lemniscular octaphyrin. Org. Lett..

[106] Katzenelson O., Edelstein J., Avnir D. (2000). Quantitative chirality of helicenes. Tetrahedron Asymmetry.

[107] Okumura T., Tani Y., Miyake K., Yokoyama Y. (2007). Chiral helicenoid diarylethene with large change in specific optical rotation by photochromism. J. Org. Chem..

[108] Wigglesworth T.J., Sud D., Norsten T.B., Lekhi V.S., Branda N.R. (2005). Chiral discrimination in photochromic helicenes. J. Am. Chem. Soc..

[109] Wang Z.Y., Todd E.K., Meng X.S., Gao J.P. (2005). Dual modulation of a molecular switch with exceptional chiroptical properties. J. Am. Chem. Soc..

[110] Eyring H. (1935). The activated complex and the absolute rate of chemical reactions. Chem. Rev..

[111] Frisch M.J., Trucks G.W., Schlegel H.B., Scuseria G.E., Robb M.A., Cheeseman J.R., Scalmani G., Barone V., Mennucci B., Petersson G.A., Nakatsuji H., Caricato M., Li X., Hratchian H.P., Izmaylov A.F., Bloino J., Zheng G., Sonnenberg J.L., Hada M., Ehara M., Toyota K., Fukuda R., Hasegawa J., Ishida M., Nakajima T., Honda Y., Kitao O., Nakai H., Vreven T., Montgomery J.A., Peralta J.E., Ogliaro F., M. Bearpark M., Heyd J.J., Brothers E., Kudin K.N., Staroverov V.N., Kobayashi R., Normand J., Raghavachari K., Rendell A., Burant J.C., Iyengar S.S., Tomasi J., Cossi M., Rega N., Millam J.M., Klene M., Knox J.E., Cross J.B., Bakken V., Adamo C., Jaramillo J., Gomperts R., Stratmann R.E., Yazyev O., Austin A.J., Cammi R., Pomelli C., Ochterski J.W., Martin R.L., Morokuma K., Zakrzewski V.G., Voth G.A., Salvador P., Dannenberg J.J., Dapprich S., Daniels A.D., Farkas O., Foresman J.B., Ortiz J.V., Cioslowski J., Fox D.J. (2009). Gaussian 09, Revision A.02.

[112] Ma X., Wang Q., Qu D., Xu Y., Ji F., Tian H. (2007). A light-driven pseudo[4]rotaxane encoded by induced circular dichroism in a hydrogel. Adv. Funct. Mater..

[113] Kobayashi N., Higashi R., Titeca B.C., Lamote F., Ceulemans A. (1999). Substituent-induced circular dichroism in phthalocyanines. J. Am. Chem. Soc..

[114] Zhao Y., Truhlar D.G. (2008). The M06 suite of density functionals for main group thermochemistry, thermochemical kinetics, noncovalent interactions, excited states, and transition elements: Two new functionals and systematic testing of four M06-class functionals and 12 other functional. Theor. Chem. Account..

